# Cortisol response to psychosocial stress, mental distress, fatigue and quality of life in coronary artery disease patients

**DOI:** 10.1038/s41598-022-23712-w

**Published:** 2022-11-12

**Authors:** Julija Gecaite-Stonciene, Brian M. Hughes, Nijole Kazukauskiene, Adomas Bunevicius, Julius Burkauskas, Julius Neverauskas, Marcella Bellani, Narseta Mickuviene

**Affiliations:** 1 Laboratory of Behavioural Medicine, Neuroscience Institute, Lithuanian University of Health Sciences, 00135 Palanga-Kaunas, Lithuania; 2 University of Galway IE, Galway, H91 TK33 Ireland; 3 Section of Psychiatry, Department of Neurosciences, Biomedicine and Movement Sciences, University of Verona, 37134 Verona, Italy

**Keywords:** Neuroscience, Physiology, Psychology, Biomarkers, Cardiology, Endocrinology

## Abstract

We aimed to explore the relationship between cortisol response to psychosocial stress, mental distress, fatigue and health related quality of life (HRQoL) in individuals with coronary artery disease (CAD) after recent acute coronary syndrome (ACS). A cross-sectional study initially included 113 subjects (88% men, 53 ± 7 years) 1–3 weeks after ACS. Cortisol response was assessed by measuring salivary cortisol during Trier Social Stress Test. Mental distress was measured with Hospital Anxiety and Depression Scale, State-Trait Anxiety Inventory, and Type D Scale-14. Fatigue symptoms were evaluated using Multidimensional Fatigue Inventory 20-items, while HRQoL was assessed with 36-Item Short Form Medical Outcome Questionnaire. After conducting multivariable linear regression analyses, diminished cortisol response sampled after Public speech (T3–T1, + 15 min) was significantly associated with higher anxiety symptoms (β = −0.224; p = 0.035), while diminished cortisol response sampled after preparation time (T2–T1, + 10 min) was significantly linked with the presence of Type D personality (β = −0.290; p = 0.006; β = −0.282; p = 0.008 respectively), even after controlling for confounders (i.e., sex, age, education, New York Heart Association functional class, beta-blockers and baseline levels of cortisol measures). We found that mental distress, but not fatigue and HRQoL, was linked with blunted cortisol response during anticipation time of psychosocial stress, independently of potential covariates.

## Introduction

Coronary artery disease (CAD) remains one of the leading causes of morbidity, mortality, and increased healthcare costs^1,2^. Even though instant fight-or-flight response to an acute stressor is healthy in majority of the individuals, it can act as a trigger for acute coronary syndrome (ACS), including unstable angina pectoris and myocardial infarction^3^ for those who are more at risk for developing heart related conditions. Meanwhile, prolonged psychosocial stress—often described as the accumulation of multiple stress reactions, such as declines in mental status, increases in somatic symptoms, and alterations in behavioral responses to stressors^4^—is one of the major risk factors for the development and progression of CAD^5^. Thus, it is important to specifically investigate psychophysiological responses to stress in acutely ill cardiac population. Nevertheless, the studies in this unique group are exceptionally rare.

The hypothalamic–pituitary–adrenal (HPA) axis is a central to physiological stress responses in directly stimulating the release of stress hormones, including cortisol^6^. Dysregulation of HPA axis activity might also be implicated in the pathogenesis of CAD^7^ and contribute to a health-related problems in individuals with cardiac conditions. In fact, the HPA axis disturbances can serve as a predictor of heart related conditions, including CAD. Pathological function of HPA axis activity, such as low cortisol variability, was found to be a risk factor for future development of CAD^8^. Another study by Nijm et al.^7^ found that individuals with CAD showed altered cortisol response to acute stressor in comparison to healthy controls. Moreover, in those with CAD, dysfunctional HPA axis response was linked with the failure to contain inflammatory activity^7^. A recent study by Aladio et al.^9^ investigated 236 individuals with CAD following recent ACS and found that those who deceased during hospitalization had higher cortisol levels at admission. In another study, Nijm et al.^7^ investigated 30 individuals with CAD, findings disturbances of HPA axis functioning. Specifically, individuals with CAD showed blunted cortisol response during psychological stress, in comparison to CAD-free individuals.

Both increased and decreased cortisol responses to psychological stress can be problematic. Specifically, blunted cortisol response to emotional stress have been found in various mental health conditions^10–12^ as well as linked with adverse health outcomes^13,14^, suggesting that both exaggerated as well as diminished cortisol responses to psychological stress signal dysregulation of mechanisms related to stress reactivity^13^. It is suggested that the phenomenon of hypercortisolism may befall as a result of a prolonged period of hyperactivity of HPA-axis due to constant chronic stress^15^.

Mental distress, including depressive and anxiety symptoms together with trait anxiety and Type D (or ‘Distressed’) personality are considered as psychosocial risk factors in the aetiology and pathogenesis of cardiovascular disorders^16–18^. Type D personality, which is highly common among individuals with CAD^19^, is manifested as a high tendency toward negative affectivity and social inhibition^20^.

Some investigators have examined whether mental distress variables impact on psychophysiological stress pathways by influencing HPA activity. In terms of cortisol response to stress, a study by Jezova et al.^21^ detected blunted cortisol responses in individuals who had high levels of anxiety. Another study by Waller et al.^14^ linked blunted cortisol responses with depression in CAD patients. Further, in a study by Whitehead et al.^22^, cortisol awakening responses were found to be associated with the presence of Type D personality in people who recently had ACS, echoing previous research that had linked Type D personality to prolonged dysregulation of the HPA axis function in this population^23^. Finally, in terms of cortisol response during mental stress in these patients, a decade ago Brydon et al.^24^ found blunted cortisol response to be associated with trait hostility, a characteristic commonly related to Type D personality^20,25^.

Fatigue, defined as the subjective experience of persevering mental and physical exhaustion^26–28^, is known to be problematic in those with heart related conditions^29,30^. Several studies in individuals with CAD suggested links between subjectively perceived stress and fatigue^31,32^. In terms of objectively observed psychophysiological stress markers, in our recent study^33^ we found diminished cardiovascular reactivity to stress linked with higher levels of fatigue during anticipation of mental stress challenge, suggesting fatigue as a possible variable contributing to dysregulated psychophysiological response to stress in those after recent ACS. Nevertheless, even though the interplay between cortisol stress reactivity and fatigue has been extensively studied in persons with chronic fatigue syndrome^34–36^, there was no study that explored cortisol response to psychosocial stress and its interplay with fatigue in individuals with ACS.

Dysregulated HPA activity is known to be linked to worse health-related quality of life (HRQoL)^37^, which is a construct comprised of subjective health outcomes, such as psychological and physical well-being^38,39^. The interactions between HPA axis activity during stress and HRQoL have been investigated in diverse clinical populations, including individuals with psychiatric^40^, oncological^41^, and gynecological^42^ conditions. However, individuals with ACS have received less attention in this regard, even though prolonged stress and worse HRQoL contribute significantly to the development and progression of CAD^43,44^.

Overall, the pathophysiological mechanisms underlying emotional triggering in those with CAD after ACS are still poorly understood, even though the dysregulation of HPA axis activity might be a potential mechanism linking psychological and health related factors with the presence of CAD. Thus, this study had the overall exploratory aim to investigate the relationship between cortisol response to psychosocial stress, mental distress, fatigue and HRQoL in individuals with CAD after recent ACS, while controlling for possible covariates. Considering the results from previous studies^14,21,24^, we hypothesized that mental distress (i.e. presence of Type D personality and high trait anxiety as well as higher depressive, and anxiety symptoms) will be associated with lower cortisol response during TSST after comprehensively controlling for possible confounders.

## Methods

### Study participants

For this cross-sectional study, individuals with CAD were recruited within 2–4 days of admission to the inpatient cardiac rehabilitation clinic at the Lithuanian University of Health Sciences, Neuroscience Institute, Hospital Palangos Klinika, Palanga, Lithuania. All participants were admitted to the clinic within one week following treatment for ACS (i.e. myocardial infarction or angina pectoris). Our inclusion criteria were: (1) diagnosis of acute myocardial infarction or unstable angina pectoris, (2) participation in cardiac rehabilitation program, (3) able to hear, speak and read in Lithuanian, and (4) signed informed consent.

Participant with arrhythmic disorder and/or after implantation of cardioverter defibrillator and with other cardiac defects needing surgical intervention were not invited to participate in the study. A total of 176 patients met the initial inclusion criteria.

Further, exclusion criteria were then applied, including: (1) cognitive and communicative disabilities 12 (6.8%), (2) severe comorbidities, such as cancer, kidney failure and motor function impairment, 6 (3.4%), (3) unstable cardiovascular condition 22 (12.5%), (4) age above eighty years 13 (7.4%) and (5) unwillingness to participate in the study 10 (5.7%). In total, 63 (35.8%) individuals were excluded from the study. The final sample of study participants consisted of 113 individuals with CAD after ACS (87% men, mean age of 53 ± 8). All participants were subjected to standard evaluation and treatment for the secondary prevention of CAD according to the existing guidelines^43,45,46^. Some parts of the methods and first preliminary results on cardiovascular reactivity to psychosocial stress was first described in our earlier study with 116 CAD patients^47^ (Detailed flowchart of participants is included as Appendix 2).

### Study procedure

Within two days of admission to the rehabilitation program and after providing written consent, subjects were prospectively evaluated for socio-demographic and clinical factors that included age, gender, education, marital status, New York Heart Association (NYHA) functional class^48^, presence of arterial hypertension (AH), obesity (body mass index [BMI] > 30 kg/m2), and smoking habits^46^. Individuals with CAD were also evaluated for medication use, including beta-blockers, nitrates, angiotensin-converting enzyme inhibitors, diuretics and benzodiazepines. Baseline demographic and clinical data were obtained from the medical records.

During the same time study participants completed a battery of self-report questionnaires for evaluation of subjective fatigue levels, symptoms of depression and anxiety, trait and state anxiety, Type D personality, and HRQoL. Finally, within ten days of admission, all study participants underwent Trier Social Stress Test (TSST). Cortisol response was assessed by measuring salivary cortisol at baseline and following exposure to the TSST.

All procedures and experimental protocols conducted in the current research involving human subjects followed the ethical principles and were approved by the Ethics Committee for Biomedical Research at Lithuanian University of Health Sciences, Kaunas, Lithuania (Protocol No. BE-2-21; P1-38/2007; P2-38/2007) and conformed to the principles outlined in the Declaration of Helsinki. Informed consent was attained from each participant agreeing to be enrolled in the study.

### Measures

*Hospital Anxiety and Depression Scale* Anxiety and depressive symptoms were measured using a well-validated Lithuanian version^49,50^ of the Hospital Anxiety and Depression Scale (HADS)^51^. The HADS has 14 self-reported questions that assess the intensity of anxiety and depression symptoms during the last two weeks. It is based on a four-point (0–3) response category. The total score ranges from 0 to 21 for both subscales, with the higher scores indicating more severe symptoms. Scores of eight or more indicate the presence of serious symptoms. In Lithuanian individuals with CAD, the HADS have sufficient psychometric characteristics^52^ and is commonly used in this specific population worldwide^53^. In our study, the HADS showed adequate internal consistency: HADS-A Cronbach’s α = 0.82 and HADS-D Cronbach’s α = 0.72.

*State-Trait Anxiety Inventory* The State-Trait Anxiety Inventory is comprised of two self-reported questionnaires: Trait version (STAI-T) was developed to evaluate a stable tendency to experience anxiety and predispositions to experience stressful situations as threatening, while State version (STAI-S) was created to measure situational anxiety, defining how the participant feels at the current moment^54^. Each questionnaire consists of 20 items that are based on 4-point (1 to 4) Likert scale. The higher score indicates higher level of trait or state anxiety. Scores on each scale that are ≥ 30 points indicate moderate, while scores ≥ 45 determines severe anxiety^54^. In Lithuanian individuals with CAD, the STAI-T and STAI-S has shown adequate psychometric properties^55^. In the current study, good internal consistency of STAI-T with Cronbach’s α = 0.87, and STAI-S with Cronbach’s α = 0.93 was detected.

*Type D Scale-14* The Type D Scale-14 (DS14)^20^ was used to evaluate distressed or Type D personality trait and includes two seven-item subscales measuring stable personality traits of negative affectivity (NA) and social inhibition (SI). A score of ten or greater on both subscales indicates Type D personality. Previous studies in CAD population^56^, as well as our current study report adequate psychometric characteristics of the DS14 with Cronbach’s α = 0.79.

*Multidimensional Fatigue Inventory, 20-items* Fatigue severity was measured by employing subscales from the Multidimensional Fatigue Inventory, 20-items (MFI-20)^50,57,58^. The MFI-20 covers five subscales: (1) general fatigue, (2) physical fatigue, (3) mental fatigue, (4) reduced activity, and (5) reduced motivation. Each domain consists of four items with possible answers on a five-point Likert scale (1 = “yes, that is true”; 5 = “no, that is not true”)^33^. The domain of General fatigue is composed of the general statements about fatigue and reduced functioning, covering physical as well as psychological aspects of fatigue. Physical fatigue concerns physical feelings related to fatigue. Mental fatigue is linked to cognitive functioning, such as difficulty concentrating. The reduced activity subscale assesses the influence of psychological and physical factors on one's level of activity. The low motivation subscale reflects a lack of motivation to start an activity. The total score ranges from 4 to 20 on each subscale, and 20 to 100 for total fatigue score with higher score indicating higher fatigue levels. Cronbach ‘s α coefficients of almost all MFI-20 subscales ranged from 0.63 to 0.93.

*36-Item Short Form Medical Outcome Questionnaire* The 36-Item Short Form Medical Outcome Questionnaire (SF-36) evaluates eight major domains of HRQoL including physical function, role limitations due to physical problems, role limitations due to emotional problems, social functioning, mental health, vitality, pain, and general health perception. Each of the eight SF-36 subscales are scored on a scale from 0 to 100, with higher scores indicating better HRQoL^59^. In the current study, Cronbach ‘s coefficients α of almost all SF-36 subscales ranged from 0.71 to 0.83, except for the social functioning subscale with Cronbach’s α = 0.45. Several authors suggest that such a coefficient might tentatively be accepted if the subscale is comprised of few items^60^, but the results relating to this subscale should be interpreted with caution. The SF-36 was validated in Lithuania^61^ and previous studies have reported similar internal consistency of Lithuanian translation of the SF-36 in individuals with CAD^62,63^.

*Trier Social Stress Test* To evaluated cortisol response during acute psychosocial stress in laboratory settings, we used the TSST^64^, which is considered a golden standard for evaluating the neurobiology of acute stress^65^. We followed the standard TSST protocol^66–68^, with the exception of an adjustment to the arithmetic task^69^. Instead of using serial subtraction, we employed the Paced Auditory Serial Addition Test (PASAT)^70–72^.

Experimental sessions of TSST were conducted between 2:30 and 3:30 PM and were comprised of several phases. In the beginning, participants were given time to rest (Baseline rest, 10 min.), after which they were exposed to initial anticipatory stress in the form of instructions for the first task (Task instructions, 5 min.). Participants then underwent Preparation time (5 min.), after which they had to present themselves at the simulated job interview (Public Speech, 5 min.) in front of a committee comprised of trained researchers. Then, participants underwent the Arithmetic task (8 min.) as a second stressor, after which they sat for a final Recovery period (15 min.) (Appendix 1). There were no specific interventions to equalize participants’ blood sugar levels. However, all the participants followed similar diet provided at the cardiovascular rehabilitation.

In terms of the modified arithmetic task, the PASAT involved voice recording listing numbers from 1 to 9. Study patients had to add each number presented on the voice recording to the immediately preceding number and to say the answer aloud. PASAT was comprised of four series of numbers, with progressively shorter inter-digit time intervals. Two lab assistants and a licensed medical psychologist participated in the TSST administration. After the TSST, there was a 15 min recovery time during which study patients could relax quietly by themselves. Subjects received a debriefing about the tasks' goals and were provided with the answers to their questions.

Saliva samples were obtained after Baseline rest at time + 0 min. (T1), Preparation time at time + 10 min. (T2), Public speech at time + 15 min. (T3), Arithmetic task at time + 23 min. (T4) and Recovery period at time + 38 min. (T5) for subsequent analysis of cortisol concentration (Appendix 1). As a baseline cortisol measure, we considered T1, which was taken after 10 min rest, based on the original study of TSST by Kirschbaum et al.^64^ Saliva samples were obtained using “Salivette” (Sarstedt, Inc.) swabs (which the participant chewed for 30–90 s until it was filled with 0.5–1.0 mL of saliva. Samples were then stored at − 70 °C and cortisol levels were determined in a licensed laboratory using commercial enzyme kits. Test samples were taken by a registered nurse. Due to the time lag in cortisol responsiveness^73^, it was difficult to differentiate the time point when cortisol concentration reflected the actual response to the stressor. Based on Miller et al.^74^, we chose time point of + 25 min (which was the closest approximation to the time when T4 was taken) after baseline to differentiate responders vs. non-responders to TSST (T4–T1), and thus timepoint when cortisol stress reactivity occurred.

Due to the same time delay of cortisol stress responsivity, there was also a methodological challenge to determine the cortisol concentration during the recovery. In this study we chose to take early recovery sample of cortisol concentration 15 min after the active TSST phases, which is similar to earlier studies^75^ and close to original Kirschbaum et al. study^64^. Yet it is important to note that cortisol concentration samples taken during the early recovery phases may still be affected by the active stressors during TSST^64^.

The value of cortisol response (Δnmol/l) was derived by subtracting the cortisol value sampled during Baseline rest from the cortisol value taken during the specific TSST phase.

Due to the physical safety, the participants were monitored by the cardiologist during the TSST. This laboratory experiment was terminated earlier if the study patient had maladaptive exaggerated cardiovascular reactivity (i.e. a rise of blood pressure ≥ 210/115 mmHg)^76^.

*Visual Analogue Scales* After the TSST, study participants were debriefed about the purpose of the study and the subjective measure of perceived efforts and perceived difficulty of the TSST tasks were collected by using Visual Analogue Scales (VAS). The scales ranged from 0 (maximum difficulty/efforts) to 100 (minimum difficulty/efforts). VAS were chosen based on its applicability in experimental clinical studies^77^ and common use in combination with TSST^78^.

### Statistical analysis

SPSS Statistics for Windows, Version 22.0.0.0 (IBM SPSS Statistics for Windows, Version 22.0. Armonk, NY: IBM Corp) was employed for statistical analysis.

Before further statistical analysis, we determined possible outliers. Univariate outliers were identified as z-scores > 2.26 (p < 0.001, two tailed). Multivariate outliers were determined by using Mahalanobis distances, by using chi-square cut-off point (p < 0.001). In total, 15 outliers were eliminated from the further study, including 14 univariate outliers and a 1 multivariate outlier, remaining 98 participants for the final analysis.

To compare sociodemographic and clinical characteristics, mental distress, fatigue, HRQoL and cortisol responses during TSST, we used two-tailed Student’s t test or Mann Whitney U test for continuous variables and Fisher’s χ2 test for categorical variables.

To determine whether TSST was a valid instrument to induce acute psychosocial stress, linear mixed models were used. To determine the links between cortisol response to TSST and mental distress, fatigue, HRQoL as well as sociodemographic and clinical characteristics, series of univariate regression analyses were performed. Due to the large number of independent variables, Benjamini–Hochberg adjustment for multiple comparisons was employed, setting a critical value for false discovery rate of 0.10^79^.

We used univariate regression analysis to evaluate the links between cortisol response to TSST and mental distress, HRQoL and fatigue as well as sociodemographic and clinical characteristics (as possible covariates in the further analysis). Finally, multivariable linear regression analyses were used to evaluate links between mental distress, fatigue, HRQoL and cortisol response to TSST, while controlling for possible covariates, which were chosen based on the results of univariate analysis and previous literature. This statistical method was chosen due to the different number of participants in the TSST phases (as a result maladaptive exaggerated cardiovascular reactivity, which led to early termination of TSST) (Appendix 2). Analysis for multicollinearity showed adequate results (variance inflation factor values < 4).

## Results

As presented in Table 1, participants’ mean age was 53 years (SD = 7.2) and were predominantly males (87.8%) with mostly high school degrees (50.0%) and College/University degrees (50.0%). In total, 43.9% (n = 43) of the participants met the criteria for obesity and had either past or present experience of nicotine use (57.1%). Most of the participants were admitted to the hospital due to acute myocardial infarction (73.5%), while the rest of them met the criteria for unstable angina pectoris (26.5%). According to the NYHA functional classification system, most participants met the criteria for Class II (85.7%), representing limitation of physical activity but comfort at rest. The majority had a comorbid diagnosis of AH (86.7%) and all were under the pharmacological treatment. All participants were within the normal range of cognitive functioning, and were able to understand the instructions of the scales and complete the TSST. Around one third (27.6%) of participants met the criteria for Type D personality and had significant anxiety symptoms (25.5%), while 7.1% of participants had significant depressive symptoms.

**Table 1:** Descriptive information of study participants.

	Total group
	N = 98
Age, mean ± SD	52.92 ± 7.17
**Sex, n(%)**	
Men	86 (87.8)
Women	12 (12.2)
**Education, n(%)**	
High school	49 (50.0%)
College/university degree	49 (50.0%)
**Diagnosis, n(%)**	
Unstable angina pectoris	26 (26.5%)
Acute myocardial infarction	72 (73.5%)
**Medication use, n(%)**	
Nitrates	5 (5.1%)
ACE inhibitors	82 (83.7%)
Diuretics	9 (9.2%)
Betablockers	81 (82.7%)
Benzodiazepines	5 (5.1%)
**New York Heart Association functional class, n(%)**	
I	9 (9.2%)
II	84 (85.7%)
III	5 (5.1%)
Obesity (body mass index > 30 kg/m^2^), n(%)	43 (43.9%)
Arterial hypertension, n(%)	85 (86.7%)
Nicotine use (smoking currently/in the past), n (%)	56 (57.1%)
Presence of type D personality (DS14), n(%)	27 (27.6%)
**Anxiety symptoms (Hospital Anxiety and Depression scale), n(%)**	
Total score < 8	73 (74.5%)
Total score ≥ 8	25 (25.5%)
**Depressive symptoms (Hospital Anxiety and Depression scale), n(%)**	
Total score < 8	91 (92.9%)
Total score ≥ 8	7 (7.1%)
State anxiety (The State-Trait Anxiety Inventory), n(%)	
Total score < 45	84 (85.7%)
Total score ≥ 45	14 (14.3%)
**Trait anxiety (The State-Trait Anxiety Inventory), n(%)**	
Total score < 45	60 (61.2%)
Total score ≥ 45	38 (38.8%)
Health related quality of life (36-Item Short Form Survey) scores:	
Physical functioning, mean ± SD	73.93 ± 16.24
Role limitation due to physical problems, median (IQR)	25.00 (0; 75.0)
Role limitation due to emotional problems, median (IQR)	66.67 (33.33; 100)
Social functioning, mean ± SD	72.56 ± 21.24
Mental health, mean ± SD	71.74 ± 16.96
Vitality, mean ± SD	63.52 ± 17.95
Pain, mean ± SD	55.51 ± 24.25
General health perception, mean ± SD	59.49 ± 16.94
**Fatigue (Multidimensional Fatigue Inventor-20) scores**	
Global fatigue, mean ± SD	9.85 ± 4.01
Physical fatigue, mean ± SD	11.06 ± 4.65
Activity reduction, mean ± SD	11.87 ± 4.28
Motivation reduction, mean ± SD	8.60 ± 3.28
Mental fatigue, mean ± SD	9.29 ± 3.95
Total Fatigue score, mean ± SD	50.66 ± 16.74
Cortisol Measures (nmol/l) during Trier Social Stress Test	
Baseline rest, mean ± SD	5.84 ± 2.20
Task instruction, mean ± SD	6.50 ± 2.64
Public speech, mean ± SD	6.95 ± 2.97
Arithmetic task, mean ± SD	9.67 ± 4.52
Recovery time mean ± SD	15.02 ± 8.96
Cortisol’s response* (nmol/l) during Trier Social Stress Test	
Task instruction, mean ± SD	0.89 ± 2.20
Public speech, mean ± SD	1.36 ± 2.36
Arithmetic task, mean ± SD	4.06 ± 4.73
Recovery time, mean ± SD	8.67 ± 8.37
**Category based on cortisol response**	
Responders (*Δ* > 1.5 nmol*/l*), n(%)	44 (44.9%)
Non-Responders (*Δ* < 1.5 nmol*/l*), n(%)	50 (54.1%)

*The value of cortisol response (Δnmol/l) was derived by subtracting the cortisol value during Baseline rest from the cortisol value during the specific Trier Social Stress Test phase.

**Figure 1:**
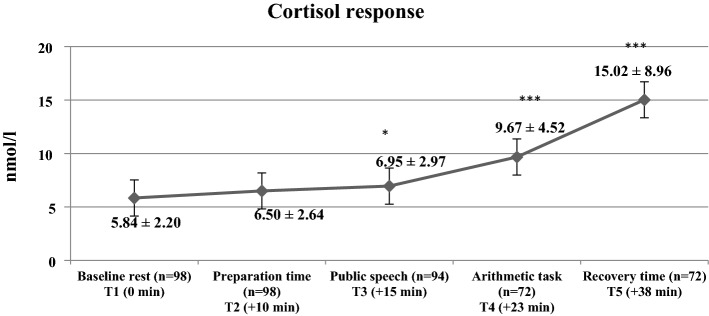
Descriptive statistics of cortisol measures and comparison with baseline cortisol measures during Trier Social Stress Test in the study participants. (*p < 0.05; ***p < 0.001 as compared with baseline rest.).

As depicted in Fig. 1, after conducting linear mixed models, the significant increase in cortisol measures as response to TSST was observed (F[4;429] = 50.72, p < 0.001, η^2^ = 0.321).

As a result of exaggerated cardiovascular reactivity (a rise of blood pressure ≥ 210/115 mmHg), 4 (4%) study patients did not proceed with stress evoking tasks of TSST during the task of Public speech, while only 72 participants (73%) partaken TSST Arithmetic task, remaining 26 participants (27%) who did not completed the Arithmetic task (Appendix 2).

Univariate analysis (Table 2) indicated that higher anxiety symptoms were associated with diminished cortisol response to TSST (T3–T1) sampled after simulated job interview (p < 0.01), while the presence of Type-D personality was associated with diminished cortisol response to TSST (T2–T1) sampled during preparation time (T2–T1). Cortisol response was also significantly associated with HRQoL (domains of vitality and social functioning) (p < 0.05). However, after correction for multiple comparisons, HRQoL could not be included in the further analysis.

**Table 2:** The links between cortisol response to Trier Social Stress Test (TSST) in 6 phases and sociodemographic and clinical factors as well as mental distress, fatigue and health related quality of life (HRQoL) in study patients (n = 98).

	Preparation time (n = 98) (Cortisol response, T2–T1, Δnmol/l)	Public Speech (n = 94) (Cortisol response, T3–T1, Δnmol/l)	Arithmetic task (n = 72) (Cortisol response, T4–T1, Δnmol/l)	Recovery time (n = 72) (Cortisol response, T5–T1, Δnmol/l)
**Sociodemographic and clinical characteristics**				
Sex	0.050 (0.625)	− 0.050 (0.633)	0.016 (0.895)	0.053 (0.657)
Age	0.061 (0.551)	− 0.063 (0.544)	− 0.212 (0.074)	0.098 (0.414)
Body mass index	0.042 (0.683)	0.021 (0.838)	0.097 (0.419)	− 0.120 (0.316)
Education	− 0.116 (0.254)	0.096 (0.358)	− 0.016 (0.894)	− 0.012 (0.992)
NYHA functional class	− 0.094 (0.358)	− 0.138 (0.185)	− 0.108 (0.365)	− 0.039 (0.747)
Arterial hypertension	− 0.030 (0.770)	0.091 (0.385)	0.035 (0.769)	− 0.085 (0.479)
Smoking history	0.065 (0.524)	− 0.029 (0.782)	− 0.004 (0.972)	− 0.166 (0.162)
**Medication use**				
Nitrates	− 0.107 (0.295)	− 0.104 (0.318)	0.039 (0.746)	0.198 (0.096)
ACE inhibitors	0.060 (0.559)	0.060 (0.563)	0.106 (0.375)	0.088 (0.462)
Diuretics	− 0.013 (0.897)	− 0.101 (0.331)	− 0.048 (0.691)	− 0.082 (0.492)
Beta-blockers	− 0.121 (0.236)	**0.212 (0.041)**	**0.344 (0.003)**	**0.302 (0.010)**
Benzodiazepines	− 0.037 (0.714)	− 0.070 (0.504)	0.050 (0.676)	0.226 (0.056)
**Mental distress, HADS**				
Depressive symptoms, HADS-D	− 0.148 (0.146)	− 0.109 (0.295)	− 0.071 (0.554)	− 0.136 (0.255)
Anxiety symptoms, HADS-A	− 0.134 (0.187)	− **0.296 (0.004)**	− 0.152 (0.202)	− 0.131 (0.274)
State anxiety, STAI-S	− 0.041 (0.692)	− 0.135 (0.051)	− 0.000 (0.997)	− 0.038 (0.751)
Trait anxiety, STAI-T	0.028 (0.787)	0.001 (0.996)	0.017 (0.890)	0.056 (0.643)
Presence of Type D personality, DS14	− **0.290 (0.004)**	− 0.143 (0.168)	− 0.145 (0.224)	− 0.165 (0.166)
**Fatigue, MFI-20**				
Global fatigue	− 0.011 (0.912)	− 0.009 (0.932)	− 0.028 (0.818)	− 0.081 (0.497)
Physical fatigue	− 0.037 (0.718)	− 0.183 (0.077)	− 0.134 (0.263)	− 0.125 (0.296)
Activity reduction	− 0.109 (0.284)	− 0.169 (0.104)	− 0.154 (0.195)	− 0.146 (0.221)
Motivation reduction	− 0.084 (0.411)	− 0.035 (0.738)	− 0.068 (0.573)	− 0.027 (0.821)
Mental fatigue	− 0.016 (0.879)	0.003 (0.979)	− 0.084 (0.482)	− 0.147 (0.219)
Total fatigue score	− 0.061 (0.550)	− 0.102 (0.326)	− 0.115 (0.338)	− 0.128 (0.283)
**Health related Quality of Life (HRQoL), SF-36**				
Physical functioning	0.154 (0.131)	0.152 (0.143)	0.066 (0.582)	0.106 (0.373)
Role limitation due to physical problems	− 0.086 (0.402)	− 0.075 (0.471)	− 0.149 (0.212)	− 0.062 (0.607)
Role limitation due to emotional problems	− 0.070 (0.495)	0.152 (0.145)	0.051 (0.672)	0.051 (0.670)
Social functioning	− 0.061 (0.548)	0.027 (0.797)	− 0.116 (0.330)	− 0.028 (0.815)
Mental health	− 0.140 (0.168)	0.189 (0.068)	0.068 (0.568)	0.105 (0.379)
Vitality	− 0.106 (0.298)	**0.234 (0.023)**	0.103 (0.389)	0.129 (0.282)
Pain	− 0.125 (0.220)	0.140 (0.177)	0.092 (0.441)	0.183 (0.124)
General health perception	0.019 (0.850)	**0.207 (0.046)**	0.015 (0.900)	− 0.0210 (0.863)
Perceived difficulty (VAS)	− 0.112 (0.287)	0.014 (0.899)	− 0.154 (0.211)	− 0.008 (0.949)
Perceived efforts (VAS)	0.066 (0.532)	0.005 (0.960)	− 0.085 (0.493)	0.214 (0.080)

Univariate linear regression analyses, r’s (p).

*MFI-20* Multidimensional Fatigue Inventory 20-items, *SF-36* 36-Item Short Form Medical Outcome; Questionnaire, *NYHA* New York Heart Association, *HADS-A* Hospital Anxiety and Depression Scale, anxiety symptoms subscale, *HADS-D* Hospital Anxiety and Depression Scale, depressive symptoms subscale, *DS14* Type D Scale-14, *VAS* Visual Analogue Scale, *ACE* Angiotensin-converting enzyme.

Sex (male [1]; female [2]), education (high school [1]; college/university degree [2]), NYHA functional class (I-II class [1]; III class [2]), smoking (yes [0]; no [1]), medication use (yes [0]; no [1]), Type D personality (yes [0]; no [1]); Visual analog scale range 0–5.

To evaluate cortisol response (delta scores), we subtracted the averaged values of the cortisol measures (nmol/l) during Baseline rest from the averaged values during other TSST phases.

*Significant correlations (p value < .05) in bold.

After controlling for possible confounders (i.e. sex, age, education, NYHA functional class, beta-blockers and baseline levels of cortisol measures), diminished cortisol response sampled after Public speech (T3–T1, + 15 min) remained to be significantly associated with higher anxiety symptoms (β = −0.224; p = 0.035) (Table 3), while diminished cortisol response sampled after preparation time (T3–T1, + 10 min) remained to be significantly linked with the presence of Type D personality (β = −0.290; p = 0.006) (Table 4). There were no associations observed between cortisol measures sampled after baseline rest (T1, 0 min) and mental distress, HRQoL and fatigue.

**Table 3:** Multivariable linear regression model, examining anxiety symptoms and its links with cortisol response to Trier Social Stress Test, while controlling for possible confounders.

Predictors	Public speech (n = 94) Cortisol response (Δnmol/l)	
	β	p
Anxiety symptoms, HADS-A	** − 0.224**	**0.035**
Sex	− 0.025	0.814
Age	− 0.092	0.367
Education	0.135	0.208
New York Heart Association functional class	− 0.125	0.224
Beta-blockers	0.155	0.144
Baseline cortisol measures	− 0.113	0.266
F (df, df)	2.10 (7, 85)	
P value	0.053	
R^2^	0.147	
R^2^_Adjusted_	0.077	

*Significant correlations (*p* value < .05) in bold. HADS-A, Hospital Anxiety and Depression Scale.

**Table 4:** Multivariable linear regression model, examining the presence of type D personality and its links with cortisol response to Trier Social Stress Test, while controlling for possible confounds.

Predictors	Preparation time (n = 98) Cortisol response (Δnmol/l)	
	β	p
Type D personality, DS14	** − 0.290**	**0.006**
Sex	− 0.146	0.169
Age	0.059	0.556
Education	− 0.134	0.198
New York Heart Association functional class	− 0.173	0.095
Beta-blockers	− 0.175	0.087
Baseline cortisol measures	− 0.009	0.932
F (df, df)	2.12 (7, 89)	
P value	0.050	
R^2^	0.143	
R^2^_Adjusted_	0.075	

*Significant correlations (*p* value < .05) in bold. DS14, Type D Scale-14.

## Discussion

In this study, we aimed to explore the relationship between cortisol response to psychosocial stress, mental distress, fatigue and HRQoL in individuals with CAD after ACS. It was hypothesized that mental distress (i.e., presence of Type D personality and high trait anxiety as well as higher depressive, and anxiety symptoms) would be associated with blunted cortisol response during TSST in study participants after comprehensively controlling for confounders.

The hypothesis was partly supported. Specifically, after controlling for covariates (i.e., sex, age, education, NYHA functional class, beta-blockers, and baseline levels of cortisol measures), higher anxiety symptoms were associated with diminished cortisol measures taken after mental stress challenge (time point + 15 min after baseline rest), while the presence of Type D personality was linked with diminished cortisol measures taken after the anticipatory stress (time point + 10 min after baseline rest). There were no significant links between depressive symptoms and cortisol measures taken during TSST, diverging from the results found in Waller et al.^14^ study including individuals with CAD. Our non-significant results might be partly explained by the limited number of individuals (7.1%) presenting clinically significant depressive symptoms (based on screening test HADS ≥ 8), resulting in the lack of variability of the levels in depressive symptomatology.

A vast percentage of participants were classified as non-responders (54.1%), which is a larger proportion in comparison to the research conducted with healthy controls^74^. This difference might be determined due to unique features of ACS pathophysiology.

Blunted cortisol during anticipatory stress was linked with higher anxiety symptoms, corresponding to previous results found in healthy participants^21^. Further, our study also extended the knowledge in terms of personality characteristics prone to mental distress and its relevance to HPA axis activity during psychosocial stress in ACS. While earlier study by Brydon et al.^24^ found the hostility to be linked with blunted cortisol response during mental stressor in those after ACS, our study revealed that the presence of Type D personality is similarly linked with anticipatory stress after mental stressor. The current study found no relationship between depressive symptoms and cortisol response to TSST, which is in line with earlier study conducted with cancer patients^80^.

In terms of fatigue and HRQoL the present study has found no relationships with cortisol response during psychosocial stress in individuals with CAD after recent ACS after correction for multiple comparisons. It is important to note that, to our knowledge, there were also no earlier studies investigating these variables together in cardiac populations. In contrast to our findings, similar study in breast cancer survivors found links between diminished cortisol response and higher levels of fatigue^81^. We also did not observe the relationship between mental distress, fatigue and HRQoL with the cortisol response to TSST sampled in the later stages of TSST (T5–T1, T4–T1). The current study found negative results, possibly due to difference in sociodemographic data, limited number of participants, and clinical conditions. In terms of HRQoL, the initial tendencies found between HRQoL (domains of vitality and social functioning) and diminished cortisol response in our study were partly in line with the ones found in our earlier studies^82^ examining cardiovascular reactivity to mental stress in individuals with CAD. Thus, future studies may replicate these results of HPA axis activity during psychosocial stress in a larger and more diverse sample of individuals with ACS.

It is important to note that our results showed continued stress reactivity in our study sample of individuals with CAD after ACS that did not return to the baseline after 15 min of stressful tasks (time point + 38 min after baseline rest). Due to unique HPA-axis activity and the method of saliva cortisol sampling, the time-lag of salivary cortisol response after psychological stressor has been observed in other studies as well^83^, including the original study on TSST protocol by Kirschbaum et al.^64^ in healthy controls, where the highest concentration of saliva cortisol were reported around + 40 min from the baseline. Similar saliva cortisol concentration peak in terms of timing was observed in Brydon et al. study with individuals after ACS^24^. As reported by Dickerson et al.^84^, cortisol levels peak around 21 to 40 min after acute stress, which was also reflected in our study.

Limitations of our study should be noted. The study was completed in a single clinic for cardiac rehabilitation, and thus generalizability of the findings to other cohorts might be problematic. Second, our study also did not employ a control group. Third, our study was cross-sectional, and so causal relationships could not be evaluated. Fourth, we reported only early recovery time (+ 38 min since baseline) precluding us from reporting the data on cortisol variability during late recovery period. Fifth, due to the acutely ill cardiac pathology, we have experienced significant attrition of the patients during laboratory induced stress. Sixth, we used the cortisol measure at baseline 10 min before the TSST, as originally suggested by the authors^64^. Yet, the methodologically about the best time to retrieve the baseline levels of cortisol concentration is still under debate, which is the methodological limitation of TSST itself^65^. Finally, the study was mostly based on the exploratory aim, thus similar replication studies are warranted for the future research.

Nevertheless, to our knowledge, our study was the first to comprehensively explore the interplay between mental distress, fatigue, HRQoL and cortisol response during psychosocial stress in individuals after ACS and one of the few reporting experimental laboratory-induced endocrine measures in those with this particular population.

To conclude, in the current study we found that higher mental distress, but not fatigue and HRQoL, was linked with blunted cortisol response during early phases of psychosocial stress challenge, even when potential covariates were considered. Due to explorative nature of the study and number of limitations, further replication of similar studies in individuals after ACS are warranted. Future studies are encouraged to further understand the neuroendocrinological mechanisms of psychophysiological stress responses and its interplay with psychological and health related characteristics often burdensome in those with CAD, while considering both heightened and diminished responses as possibly problematic.

## Supplementary Information

Supplementary Information 1.

Supplementary Information 2.

## Data Availability

The dataset analysed during the current research is available from the corresponding author (J.G-S.) upon request.
