# Supplementary material for: Cortisol response to psychosocial stress, mental distress, fatigue and quality of life in coronary artery disease patients
Source: Sci Rep. 2022 Nov 12;12:19373. doi: 10.1038/s41598-022-23712-w (PMC9653469; doi:10.1038/s41598-022-23712-w)
Supplement: Supplementary file 1 — Supplementary Information 1. [file 41598_2022_23712_MOESM1_ESM.docx]

**Appendix 1.** The graphical overview of Trier Social Stress Test (TSST) used in this research.

| **Phase** | **Baseline rest** | | | | | | | ***Active component of TSST*** | | | | | | | | | | | | | | | | | | **Recovery time** | | | | | |
| --- | --- | --- | --- | --- | --- | --- | --- | --- | --- | --- | --- | --- | --- | --- | --- | --- | --- | --- | --- | --- | --- | --- | --- | --- | --- | --- | --- | --- | --- | --- | --- |
|  |  |  |  |  |  |  |  | **Task instruction** | | | | **Speech preparation** | | | | **Speech** | | | | **Mathematical task** | | | | | |  |  |  |  |  |  |
| **Duration** | 10 min | | | | | | | 5 min | | | | 5 min | | | | 5 min | | | | 8 min | | | | | | 15 min | | | | | |
| **Sampling time point (Saliva cortisol)** |  | | | | | | **T1** |  | | | |  | | | **T2** |  | | | **T3** |  | | | | **T4** | |  | | | | | **T5** |
| **Timeline (min)** | -10 | |  | | | | 0 |  | | | +5 |  | | | +10 |  | | | +15 |  | | | | | +23 |  | | | | | +38 |
| **Timeline (min)** | -9 | -8 | | -6 | -3 | 0 | | +1 | +3 | +5 | | +5 | +8 | +10 | | +11 | +13 | +15 | | +16 | +18 | +21 | +23 | | | +24 | +26 | +29 | +31 | +33 | +38 |
