# Supplementary material for: Cortisol response to psychosocial stress, mental distress, fatigue and quality of life in coronary artery disease patients
Source: Sci Rep. 2022 Nov 12;12:19373. doi: 10.1038/s41598-022-23712-w (PMC9653469; doi:10.1038/s41598-022-23712-w)
Supplement: Supplementary file 2 — Supplementary Information 2. [file 41598_2022_23712_MOESM2_ESM.docx]

| **Invited participants** | |
| --- | --- |
| 176 | |
| **Exclusion criteria:** | |
| Severe comorbid illness | 6 (3.4%) |
| Unstable cardiovascular condition | 22 (12.5%) |
| Age above 80 | 13 (7.4%) |
| Significant communicative difficulties or cognitive impairment | 12 (6.8%) |
| Unwillingness to participate in the study | 10 (5.7%) |
| **Participants included in the study:** | |
| 113 | |
| **Final study sample after elimination of outliers:** | |
| 98 | |

**2-4 days after admission to cardiac rehabilitation:**

Sociodemographic, clinical and CAD risk factors assessed or retrieved from medical records within 2-4 days after admission by either study cardiologist or clinical psychologist from all selected study participants.

**2-4 days after admission to cardiac rehabilitation:**

Completion of self-report questionnaires for depression, anxiety, state and trait anxiety, Type D personality, quality of life, and fatigue.

**7-9 days after admission to cardiac rehabilitation:**

Trier social stress test is completed (time 14:30)

| **The reduction of study participants during Trier Social Stress Test (TSST) due to maladaptive increase in cardiovascular parameters (i.e., a rise in blood pressure ≥210/115 mmHg)** | |
| --- | --- |
| *Phases of Trier Social Stress Test (TSST):* | |
| Baseline rest | 98 (100%) |
| Task instruction | 98 (100%) |
| Preparation time | 98 (100%) |
| Public speech | 94 (95.9%) |
| Arithmetic task | 72 (73.5%) |
| Recovery time | 72 (73.5%) |

**Appendix 2.** Participants flowchart in the current study.
